# SOCIAL NETWORK BRIDGING AND COGNITIVE RESERVE IN SPECIFIC DOMAINS: BENCHMARKING TO EDUCATIONAL ATTAINMENT

**DOI:** 10.1093/geroni/igae098.4258

**Published:** 2024-12-31

**Authors:** Haosen Sun, Brea Perry

**Affiliations:** University of Nevada, Reno, Reno, Nevada, United States; Indiana University, Bloomington, Indiana, United States

## Abstract

Cognitive reserve helps delay and slow cognitive decline despite neuropathology, with diverse social networks that have rich bridging potential enhancing this reserve. However, the benefits to different cognitive domains from such networks and the relative impact compared to other known resiliency factors are unclear. This study aims to identify which domain-specific cognitive reserve benefits most from social networks with higher bridging capital, with a comparison with education. Data from 217 participants in the Social Networks in Alzheimer’s Disease (SNAD) project were analyzed. Cognitive reserve was assessed using the residual approach, comparing cognitive domain performance against brain structure metrics from MRI. Social network bridging capital was measured through network characteristics such as structural holes, diversity, weak ties, and network size. Linear regressions examined the association between bridging capital and cognitive reserve across cognitive domains, comparing the effect size to educational attainment. Network bridging capital was significantly associated with general cognitive reserve and cognitive domains such as executive function, visuospatial skills, language, and episodic memory. The impact of bridging capital was comparable to or greater than that of educational attainment in these domains. However, it did not significantly enhance cognitive reserve in attention and processing speed, where education had a stronger influence. Overall, social networks with high bridging potential are particularly beneficial for enhancing cognitive reserve in certain domains. Interventions aimed at slowing cognitive decline in these areas could leverage diverse networks, especially early in disease trajectories, constituting a late-life modifiable protective factor.

